# Ankle Fracture Detection Utilizing a Convolutional Neural Network Ensemble Implemented with a Small Sample, De Novo Training, and Multiview Incorporation

**DOI:** 10.1007/s10278-018-0167-7

**Published:** 2019-04-18

**Authors:** Gene Kitamura, Chul Y. Chung, Barry E. Moore

**Affiliations:** 1 Department of Radiology, University of Pittsburgh Medical Center (UPMC), 200 Lothrop St., Pittsburgh, PA 15213 USA; 2 Center for Research Computing, University of Pittsburgh, Pittsburgh, PA 15260 USA

**Keywords:** Machine learning, Convolutional neural network, Neural network, Deep learning, Fractures, Ankle

## Abstract

To determine whether we could train convolutional neural network (CNN) models de novo with a small dataset, a total of 596 normal and abnormal ankle cases were collected and processed. Single- and multiview models were created to determine the effect of multiple views. Data augmentation was performed during training. The Inception V3, Resnet, and Xception convolutional neural networks were constructed utilizing the Python programming language with Tensorflow as the framework. Training was performed using single radiographic views. Measured output metrics were accuracy, positive predictive value (PPV), negative predictive value (NPV), sensitivity, and specificity. Model outputs were evaluated using both one and three radiographic views. Ensembles were created from a combination of CNNs after training. A voting method was implemented to consolidate the output from the three views and model ensemble. For single radiographic views, the ensemble of all 5 models produced the best accuracy at 76%. When all three views for a single case were utilized, the ensemble of all models resulted in the best output metrics with an accuracy of 81%. Despite our small dataset size, by utilizing an ensemble of models and 3 views for each case, we achieved an accuracy of 81%, which was in line with the accuracy of other models using a much higher number of cases with pre-trained models and models which implemented manual feature extraction.

## Introduction

Several recent studies have demonstrated the utility of machine learning for fracture detection in musculoskeletal images. One study performed manual feature extraction on 145 radiographs, utilized a random forest machine learning algorithm, and achieved a fracture detection accuracy of 81% [1]. Rather than requiring manual feature engineering of the images, convolution neural networks (CNNs) allow for image evaluation with native image inputs [2]. By using a large dataset of ~ 53,000 studies and training the novel DenseNet CNN de novo, one study achieved a 97% fracture detection accuracy for the femoral neck on pelvic radiographs [3, 4].

An alternative to training a CNN de novo is to use pre-trained CNN models. There are currently many readily available open-source implementations of CNNs through frameworks such as Caffe [5]. Many of these models are pre-trained on the dataset from the ImageNet Large Scale Visual Recognition Challenge (ILSVRC) containing millions of non-medical images [6]. When using pre-trained models, only the last layer or two of the CNN are re-trained on the dataset of interest, but the rest of the model is kept unchanged. These pre-trained networks have been shown to be good feature extractors, and one study achieved a fracture detection accuracy of 83% utilizing them with ~ 256,000 wrist, hand, and ankle radiographs [7]. However, pre-trained models are not always available for the newest architectures. In addition, the input channel for the pre-trained models are set at 3, so each grayscale image with 1 channel needs to be triplicated before being fed into the network. Finally, any modifications to the pre-trained network necessitate that the models be re-trained from that layer on. Therefore, as deep learning models become more specialized to medical images, the need for de novo training to incorporate more flexibility may become necessary.

All the aforementioned studies showed promising results, but required either large datasets, pre-trained models, or manual feature engineering. The purpose of our study was to determine whether we could achieve comparable accuracy with models utilizing a significantly smaller dataset and with de novo training. For our CNN architectures, 3 relatively modern models were selected: Inception V3, Resnet, and Xception. The Inception V3 Network is based on the principles of the generous use of dimensionality reduction, which resulted in a substantial decrease in computational load while increasing model performance [8]. The Resnet architecture was designed to construct deep networks that were easy to optimize and enjoyed accuracy gains with increasing depth [9]. Finally, the Xception network incorporated both concepts from the Inception and Resnet architectures for improved performance [10].

In addition, we know empirically that having more views available for a case increases the accuracy of a clinicians’ rendered diagnosis. However, most, if not all, machine learning algorithms evaluating fractures assessed only a single radiographic image. Therefore, we also wanted to provide objective evidence that deep learning models can increase their accuracy by having more radiographic views available for each case.

## Materials and Methods

An institutional IRB approval was obtained prior to research initialization. A total of 298 normal and 298 fractured ankle studies were identified by parsing Radiology reports. The imaging was reviewed by a board-certified radiologist and a fourth-year Radiology resident to make sure the imaging was concordant with the report. Once a case was verified, they were not excluded even if they only had one or two views. An ankle fracture was defined as a fracture of any bone visible on the study, including the proximal forefoot, midfoot, hindfoot, distal tibia, and distal fibula. No other inclusion or exclusion criteria were implemented. The images were then de-identified for Health Insurance Portability and Accountability Act of 1996 (HIPAA) compliance.

**Fig. 1:**
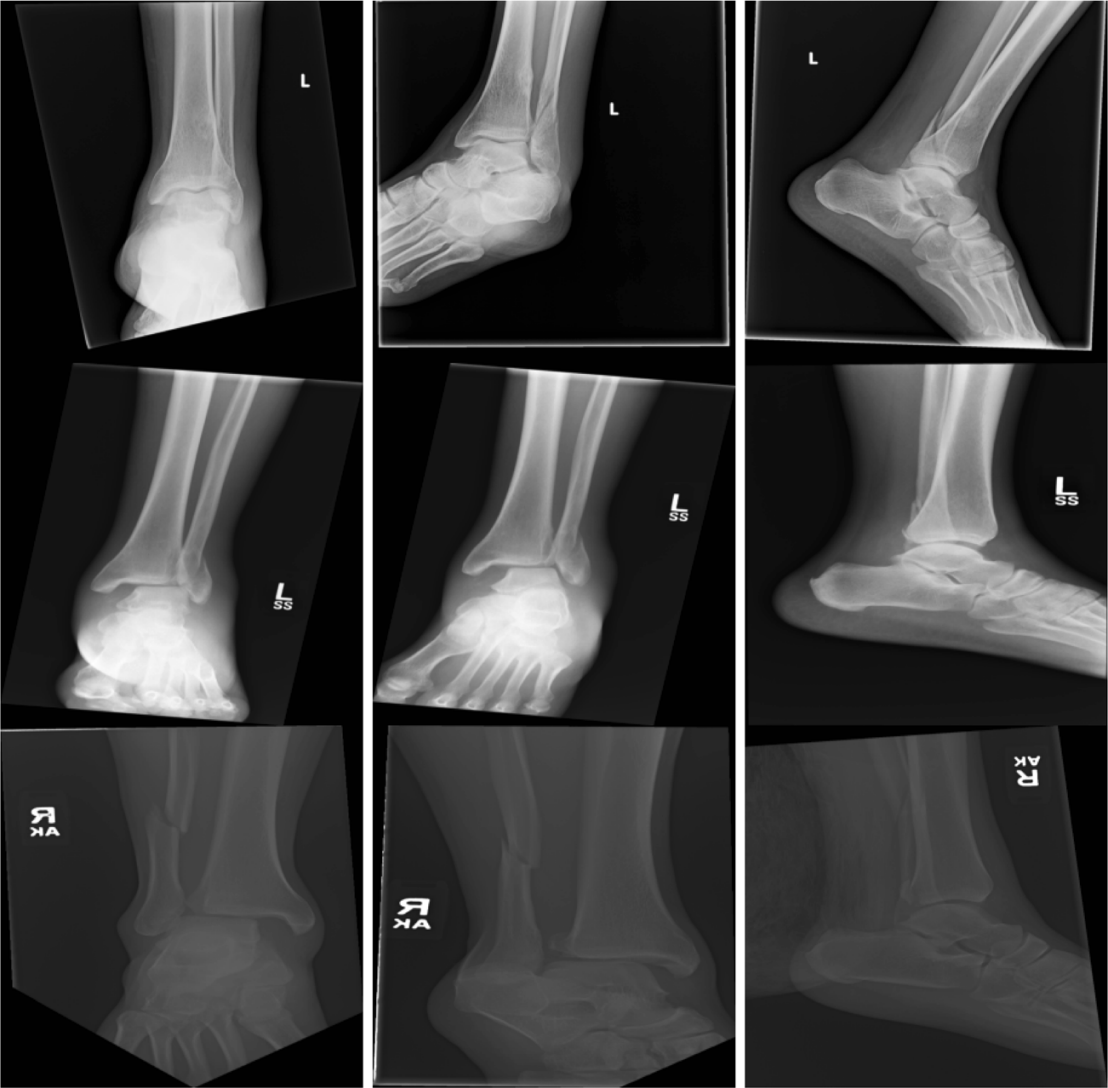
Example cases of ankle fractures. Each row represents a different patient, and the images are ordered as frontal, oblique, and lateral views from the left to the right. The first row demonstrates a minimally displaced lateral malleolar fracture with an incidental non-ossifying fibroma. The middle and last rows demonstrate trimalleolar fractures with a widened medial tibiotalar joint space

Pixel value extraction from the Digital Imaging and Communications in Medicine (DICOM) data was performed using Pydicom [11]. Each image was resized to a 300-by-300-px image with 1 grayscale channel (300 × 300 × 1) and appropriate intensity re-scaling was performed based on bits allocated using SciPy [12]. The 300-by-300 size chosen was somewhat arbitrary, but many studies on modern neural networks have chosen similar sizes [8, 10], and nearly all neural networks use a square input shape [2]. Forty normal and 40 abnormal cases with three available views for each case were extracted as the validation and test sets for a total of 240 total views. The remaining cases were utilized as the training dataset. The model was trained utilizing single radiographic views, so a total of 689 abnormal and 752 normal views for total of 1441 views were utilized for training. The uneven size of the abnormal and normal views resulted from the abnormal cases having a higher proportion of single-view and two-view imaging compared with the normal cases. Sample fracture cases are shown in Fig. 1. During the training process, data augmentation was performed to increase generalization through perturbation via random image rotation, flipping, brightness variation, and contrast alteration. Each image was standardized to a mean of 0 and standard deviation of 1, which is a common pre-processing method for deep learning models [2]. Convergence of model training was monitored using the softmax cross-entropy loss, which is a common loss function utilized for neural networks evaluating binary classifications, which in our case was normal vs fracture [2]. Models were considered converged once the loss values plateaued and no longer decreased.

A total of five different convolutional neural network architectures were constructed: Inception V3 [8], Resnet, Resnet with dropout and auxiliary tower (drop/aux) [9], Xception, and Xception with dropout and auxiliary tower (drop/aux) [10]. The Inception V3 [8] and Xception [10] networks were constructed as outlined by the original papers. For the Resnet, the 101-layer architecture was utilized, and the updated skip connection encompassing the full pre-activation identity mapping was implemented [13]. In addition, as suggested in the original paper, both a dropout layer and auxiliary tower were added to create the Resnet with drop/aux architecture to increase regularization strength. The auxiliary tower was added between the conv3 and conv4 multi-layer residual unit as described in the original paper [9]. Similar to our Resnet architecture, we also created an Xception network with both the dropout layer and auxiliary tower (drop/aux), which were suggested but only partly implemented in the original paper. The auxiliary tower was placed in the third-loop iteration of the middle flow as described in the original paper [10]. The specific layer architecture of the models is shown in Tables 1, 2, and 3.

**Table 1:** The architecture of the Inception V3 model. Each row represents a layer of the network, and the input of a particular layer is the output of the previous layer. For the inception layers, each row represents parallel sub-layers that were concatenated prior to being passed to the subsequent layer. The list of parameters in each row represents serial processes within the sub-layer. With the Inception 6 layer, the parameters within the parentheses represent additional parallel sub-layers within the serial processes. The number of times each Inception layer is repeated is prepended to each layer name

Type	Patch size/strides	Input size
Conv 1	3 × 3/2	300 × 300 × 1
Conv 2	3 × 3/1	149 × 149 × 32
Conv 3	3 × 3/1	147 × 147 × 32
Max pool 1	3 × 3/2	147 × 147 × 64
Conv 4	1 × 1/1	73 × 73 × 64
Conv 5	3 × 3/1	73 × 73 × 80
Max pool 2	3 × 3/2	71 × 71 × 192
3× Inception 1	1 × 1/1	35 × 35 × 192
	1 × 1/1, 3 × 3/1	
	1 × 1/1, 3 × 3/1, 3 × 3/1	
	Avg pool 3 × 3/1, 1 × 1/1	
Inception 2	3 × 3/2	35 × 35 × 288
	Max pool 3 × 3/2	
	1 × 1/1, 3 × 3/1, 3 × 3/2	
4× Inception 3	1 × 1/1	17 × 17 × 768
	1 × 1/1, 1 × 7/1, 7 × 1/1	
	1 × 1/1, 1 × 7/1, 7 × 1/1, 1 × 7/1, 7 × 1/1	
	Avg pool 3 × 3/1, 1 × 1/1	
Auxiliary	Avg pool 5 × 5/3, 1 × 1/1, linear, softmax	17 × 17 × 768
Inception 5	1 × 1/1, 3 × 3/2	17 × 17 × 768
	1 × 1/1, 1 × 7/1, 7 × 1/1, 3 × 3/2	
	Max pool 3 × 3/2	
2× Inception 6	1 × 1/1	8 × 8 × 1280
	1 × 1/1, (1 × 3/1, 3 × 1/1)	
	1 × 1/1, 3 × 3/1, (1 × 3/1, 3 × 1/1)	
	Avg pool 3 × 3/1, 1 × 1/1	
Avg pool	8 × 8/1	8 × 8 × 2048
Output	Dropout, linear, softmax	1 × 1 × 2048

**Table 2:** The architecture of the Resnet model. Each row represents a layer of the network, and the input of a particular layer is the output of the previous layer. Serial processes are represented as comma-separated parameters in each row. The number of times each Conv layer is repeated is prepended to each layer name. The feature map sizes are downsampled by a factor for 2 during the first iteration of Conv  3 to Conv 5. Resnet models with and without inclusion of the Auxiliary and dropout were constructed

Type	Patch size/strides	Input size
Conv 1	7 × 7/2	300 × 300 × 1
Max pool 1	3 × 3/2	147 × 147 × 64
3× Conv 2	1 × 1/1, 3 × 3/1, 1 × 1/1	74 × 74 × 64
4× Conv 3	1 × 1/1, 3 × 3/1, 1 × 1/1	74 × 74 × 256
23× Conv 4	1 × 1/1, 3 × 3/1, 1 × 1/1	37 × 37 × 512
Auxiliary	Avg pool 5 × 5/3, 1 × 1/1, linear, softmax	19 × 19 × 1024
3× Conv 5	1 × 1/1, 3 × 3/1, 1 × 1/1	19 × 19 × 1024
Avg pool	10 × 10/1	10 × 10 × 2048
Output	Dropout, linear, softmax	1 × 1 × 2048

**Table 3:** The architecture of the Xception network. The Conv 6 layer is repeated eight times. Each row represents a layer of the network. Serial processes are designated by comma-separated parameters. Xception models with and without Auxiliary and dropout were constructed

Type	Patch size/strides	Input size
Conv 1	3 × 3/2	300 × 300 × 1
Conv 2	3 × 3/1	150 × 150 × 32
Conv 3	3 × 3/1, 3 × 3/1	150 × 150 × 64
Max pool 1	3 × 3/2	150 × 150 × 128
Conv 4	3 × 3/1, 3 × 3/1	75 × 75 × 128
Max pool 2	3 × 3/2	75 × 75 × 256
Conv 5	3 × 3/1, 3 × 3/1	38 × 38 × 256
Max pool 3	3 × 3/2	38 × 38 × 728
8× Conv 6	3 × 3/1, 3 × 3/1, 3 × 3/1	19 × 19 × 728
Auxiliary	Avg pool 5 × 5/3, 1 × 1/1, linear, softmax	19 × 19 × 728
Conv 7	3 × 3/1, 3 × 3/1	19 × 19 × 728
Max pool 4	3 × 3/2	19 × 19 × 1024
Conv 8	3 × 3/1	10 × 10 × 1024
Conv 9	3 × 3/1	10 × 10 × 1536
Avg pool	10 × 10/1	10 × 10 × 2048
Output	Dropout, linear, softmax	1 × 1 × 2048

Once all the models were trained, an ensemble of models was created utilizing all possible combinations resulting in an odd number of total models. An odd number of models was necessary to prevent ties during the voting process. Specifically, 3 models were chosen from the 5 that were trained (Inception V3, Resnet, Resnet with drop/aux, Xception, and Xception with drop/aux), and the validation-test set output metrics were calculated for each model and the score was averaged. This process was performed for every possible combination of models. Ensembling was performed since previous studies have shown it to increase the performance of deep learning networks [14]. In addition, since the models were constructed to evaluate a single radiographic view, a voting method was implemented to evaluate 3 views as a single output from each model. Code is available on GitHub of the corresponding author.

The models were built utilizing the Python programming language using the Scikit-learn [15] and Tensorflow libraries [16]. Output measures included positive predictive value (PPV), negative predictive value (NPV), sensitivity, specificity, and accuracy. The models were trained on a GeForce 1080 GTX graphical processing unit (GPU) on Pitt’s Center for Researching Computing GPU cluster.

## Results

Model convergence was achieved by 2000 epochs or iterations of training taking approximately 1 day of computation per model. Training CNN models require the selection of multiple hyperparameters, which are tunable parametersin the model and are often kept close to the values determined by the original implementation. In our case, the learning rate was between 4e-6 and 6e-6, L2 decay rate was between 0.4 and 1.0, and auxiliary decay was between 0.4 and 0.9. The dropout rate was kept at 0.5.

For single radiographic views, the ensemble consisting of all five models produced the best fracture detection accuracy of 76% for the validation-test set. As expected, in essentially all cases, the output metrics demonstrated better values when all three views were utilized when compared with only a single view being evaluated (Table 4). The ensemble consisting of all five models utilizing all 3 views produced the best overall results, with output metrics greater than or equal to 80% for all parameters including accuracy, PPV, NPV, sensitivity, and specificity for the validation-test set (Table 4).

**Table 4:** The output metrics for the convolutional neural networks. Ensemble_A is comprised of a combination of all five of the convolutional neural networks. Ensemble_B consists of three models that produced the best outputs together, which in this case was the Inception V3, Resnet, and Xception with drop/aux. Output metrics were obtained using the validation-test data set

Model	Views	Accuracy	Sensitivity	Specificity	PPV	NPV
Inception V3	One	0.70	0.68	0.73	0.71	0.70
	Three	0.74	0.73	0.75	0.74	0.73
Resnet	One	0.73	0.68	0.77	0.75	0.71
	Three	0.75	0.70	0.80	0.78	0.73
Resnet with drop/aux	One	0.72	0.74	0.70	0.71	0.73
	Three	0.73	0.73	0.73	0.73	0.73
Xception	One	0.75	0.73	0.76	0.75	0.74
	Three	0.78	0.75	0.80	0.79	0.76
Xception with drop/aux	One	0.75	0.71	0.80	0.78	0.73
	Three	0.78	0.73	0.73	0.81	0.75
Ensemble_A	One	0.76	0.77	0.76	0.76	0.77
	Three	0.81	0.80	0.83	0.82	0.81
Ensemble_B	One	0.75	0.68	0.82	0.79	0.72
	Three	0.80	0.73	0.88	0.85	0.76

## Discussion

The purpose of our study was threefold: to determine whether we could use relatively small datasets and approach the accuracy of models using large datasets, to determine whether our models trained de novo approached the accuracy of pre-trained models and models requiring manual feature engineering, and to see whether we could increase the accuracy of models by using multiple views for each case. The smaller dataset issue is relevant for any researcher interested in the implementation of machine learning algorithms in the field of medical imaging. At the time of this manuscript write-up, manual data parsing, image labeling, and study de-identification take a non-trivial amount of resources in our practice setting. Furthermore, finding a clean dataset of medical images is difficult in the open-source community outside of chest radiographs [17], so we wanted to explore the lower limit of cases necessary to approach the accuracy of studies utilizing a much higher number of cases. Unfortunately, our model ensemble achieved a single radiographic view accuracy of only 76%, which was likely the result of our small sample size.

Subsequently, we implemented a voting method to consolidate the output of three separate views for a single patient into single binary output, which increased fracture detection accuracy from 76 to 81%. By using an ensemble of models and three views for each case, we were able to approach the fracture detection accuracy of pre-trained models using a large dataset of ~ 256,000 cases (83%) [7] and the model using manual feature extraction (81%) [1]. Our models were not directly trained on 3 views due to computational limitations, as the amount of data that can be loaded at once onto the GPU is limited. So, using 3 views instead of 1 view would result in significantly increased file sizes, leading to lower batch sizes and suboptimal training. In addition, training on single images retained the flexibility of evaluating a single radiographic view and potential for utilizing these trained models to evaluate other body parts, such as the pelvis.

The limitations of our study included evaluating only ankle radiographs. The body part choice was arbitrary, but we wanted to keep the study scope narrow by only focusing on one body part. Regarding accuracy, even with our three-view, ensembled model, we did not approach the 97% accuracy claimed by the study utilizing a large dataset of ~ 53,000 cases and de novo training of a DenseNet CNN [3]. As an aside, the DenseNet is an example of a novel CNN architecture for which pre-trained models were not available at the time of this study and would have necessitated de novo training [4]. Finally, the number of cases we collected ended up being suboptimal for our study; if we had instead amassed 5000–10,000 cases, our accuracy likely would have been higher.

## Conclusion

Despite our small dataset size, by utilizing an ensemble of models and 3 views for each case, we achieved an accuracy of 81%, which was in line with the accuracy of other models using a much higher number of cases with pre-trained models and models which implemented manual feature extraction.
